# SIRT1/Adenosine Monophosphate-Activated Protein Kinase α Signaling Enhances Macrophage Polarization to an Anti-inflammatory Phenotype in Rheumatoid Arthritis

**DOI:** 10.3389/fimmu.2017.01135

**Published:** 2017-09-15

**Authors:** So Youn Park, Sung Won Lee, Sang Yeob Lee, Ki Whan Hong, Sun Sik Bae, Koanhoi Kim, Chi Dae Kim

**Affiliations:** ^1^Department of Pharmacology, School of Medicine, Pusan National University, Gyeongsangnam-do, South Korea; ^2^Gene and Cell Therapy Research Center for Vessel-Associated Diseases, Pusan National University, Gyeongsangnam-do, South Korea; ^3^Department of Internal Medicine, College of Medicine, Dong-A University, Busan, South Korea

**Keywords:** rheumatoid arthritis, inflammation, macrophage polarization, M1/M2 macrophages, SIRT1, adenosine monophosphate-activated protein kinase α

## Abstract

Macrophages are crucially involved in the pathogenesis of rheumatoid arthritis (RA). Macrophages of the M1 phenotype act as pro-inflammatory mediators in synovium, whereas those of the M2 phenotype suppress inflammation and promote tissue repair. SIRT1 is a class 3 histone deacetylase with anti-inflammatory characteristics. However, the role played by SIRT1 in macrophage polarization has not been defined in RA. We investigated whether SIRT1 exerts anti-inflammatory effects by modulating M1/M2 polarization in macrophages from RA patients. In this study, SIRT1 activation promoted the phosphorylation of an adenosine monophosphate-activated protein kinase (AMPK) α/acetyl-CoA carboxylase in macrophages exposed to interleukin (IL)-4, and that this resulted in the expressions of M2 genes, including MDC, FcεRII, MrC1, and IL-10, at high levels. Furthermore, these expressions were inhibited by sirtinol (an inhibitor of SIRT1) and compound C (an inhibitor of AMPK). Moreover, SIRT1 activation downregulated LPS/interferon γ-mediated NF-κB activity by inhibiting p65 acetylation and the expression of M1 genes, such as CCL2, iNOS, IL-12 p35, and IL-12 p40. Macrophages from SIRT1 transgenic (Tg)-mice exhibited enhanced polarization of M2 phenotype macrophages and reduced polarization of M1 phenotype macrophages. In line with these observations, SIRT1-Tg mice showed less histological signs of arthritis, that is, lower TNFα and IL-1β expressions and less severe arthritis in the knee joints, compared to wild-type mice. Taken together, the study shows activation of SIRT1/AMPKα signaling exerts anti-inflammatory activities by regulating M1/M2 polarization, and thereby reduces inflammatory responses in RA. Furthermore, it suggests that SIRT1 signaling be viewed as a therapeutic target in RA.

## Introduction

Rheumatoid arthritis (RA) is a chronic inflammatory disease that can activate the immune system *via* immune cells, such as macrophages, dendritic cells, and lymphocytes. These orchestrated interactions induce the abundant productions pro-inflammatory cytokines and cellular components in RA joints, and lead to progressive joint destruction (1, 2). Macrophages in synovium exhibit a heterogeneous phenotype, and macrophages activated by various cytokines are crucial for inflammatory processes associated with development of synovitis in RA (3). Macrophages are polarized to the classically activated M1 phenotype by Th1 cytokines [LPS, interferon (IFN)γ and interleukin (IL)-12], and this phenotype polarization results in the upregulations of pro-inflammatory mediators, including TNFα, IL-1β, and MCP-1. By contrast, macrophages are polarized to the alternative M2 phenotype by Th2 cytokines (IL-4, IL-10, and IL-13), which are associated with long-term tissue repair, immunity, and the production of anti-inflammatory cytokines (4–6). Arthritic joints display an imbalance between the M1 and M2 phenotypes (7). Furthermore, signaling pathways and the activities of transcription factors are also related to macrophage polarization, for example, NF-κB, STAT1, and SOCS1 in the M1 phenotype, and STAT6, KLF4, and PPARγ in the M2 phenotype (3). Recent findings show that synovial fluids in RA patients contain high levels of M1 macrophage-derived mediators, but low levels of M2 macrophage-derived mediators (8).

Histone/protein deacetylase SIRT1 and adenosine monophosphate-activated protein kinase (AMPK) work in tandem to control cell metabolism, transcriptional gene expression, neuroprotection, and inflammation (9, 10). AMPK activation is stimulated by SIRT1 *via* the deacetylation and activation of AMPK kinase/LKB1 (11). The N-terminal domain of SIRT1 transactivates deacetylation by interacting with endogenous SIRT1 and promoting the deacetylation of K310 of the RelA/p65 subunit, and consequently reduces the activation of NF-κB activity, leading to attenuate the expressions of pro-inflammatory cytokines (12, 13). Active AMPK participates in the suppression of inflammatory responses by inhibiting inflammatory signaling, such as, the NF-κB pathway, and conversely downregulation of AMPK activity results in increased inflammation (14). Macrophages containing constitutively active AMPKα1 blocked LPS- and fatty acid-induced inflammatory mediators through SIRT1 (15), and in AMPK α1^−/−^ mice pro-inflammatory states were promoted by direct phosphorylation of NF-κB (16).

Nevertheless, the function of SIRT1 in RA remains controversial. The activity and expression of SIRT1 have been shown to be diminished in the peripheral blood mononuclear cells (PBMCs) of RA patients (17). In addition, increased SIRT1 activity has been reported to alleviate arthritis by suppressing VEGF and inhibiting synovial angiogenesis (18). On the other hand, controversial results have been reported regarding the influences of sirtuins on inflammation and apoptosis (19, 20).

Given SIRT1/AMPKα signaling has an important role in regulating macrophage polarization to an anti-inflammatory M2 phenotype, and that activation of this signaling may attenuate joint inflammation in RA, we sought to establish whether SIRT1 induces AMPKα phosphorylation and subsequent NF-κB downregulation, leading to the upregulations of M2-associated cytokines and to the downregulations of the expressions of M1-associated pro-inflammatory mediators in macrophages obtained from RA patients or SIRT1-overexpressing mice. In addition, we assessed a prevention of synovial inflammation and joint destruction in SIRT1 transgenic (Tg) mice with collagen-induced arthritis (CIA).

## Materials and Methods

### Materials

Resveratrol (RSV), LPS, compound C (CC), and Ficoll-Paque were purchased from Sigma-Aldrich (St. Louis, MO, USA). Recombinant human IL-4, recombinant human macrophage-colony-stimulating factor (M-CSF), and interferon (INF) γ were from Pepro Tech (Rocky Hill, NJ, USA). Sirtinol was obtained from Calbiochem (La Jolla, CA, USA). Antibodies specific for AMPKα, phosphor-AMPKα, phosphor-NF-κB p65 (Lys310), acetyl-NF-κB p65 (Lys310), acetyl-CoA carboxylase (ACC), and phosphor-ACC were from Cell Signaling (Danvers, MA, USA). Antibodies specific for SIRT1, NF-κB p65, ariginase-1, and Histone H1 were from Santa Cruz Biotechnology Inc. (Santa Cruz, CA, USA). Antibodies specific for TNFα and IL-1β were from Abcam (Cambridge, UK). Antibody specific for TRANCE/TNFSF11 was from R&D Systems (Minneapolis, MN, USA).

### Patient Sample

Synovial fluid was obtained during the therapeutic arthrocentesis from the affected knees of patients with RA who fulfilled the American Rheumatism Association classification criteria (1987). All patients provided informed consent, and the study was approved the Medical Ethics Committees of the Academic Medical Center (Dong-A University Hospital, Busan, South Korea).

### Cell Isolation and Cell Culture

Mononuclear cells from synovial fluid were isolated using density gradient centrifugation methods (Ficoll-Paque). Cells were incubated in RPMI with 10% FBS for 24 h, and then adherent cells were incubated in culture medium plus M-CSF (100 ng/ml) for 7 days. M1 polarization was stimulated by LPS (100 ng/ml) or INFγ (20 ng/ml), and M2 polarization was achieved by treating cells with IL-4 (20 ng/ml).

### Quantitative RT-PCR

For measurement of mRNA levels, total RNA isolation and RT-PCR were performed as previously described (21). The mRNA levels were normalized to the human ribosomal 18S gene or mouse actin. Data are analyzed using LightCycler 96 software (Roche Molecular Biochemicals). Primer sequences are provided in Table 1.

**Table 1:** Oligonucleotide sequences used for qPCR.

Human		Mouse	

Oligonucleotides, 5′–3′		Oligonucleotides, 5′–3′	
FcεRII	Forward: GGGAGAATCCAAGCAGGAC Reverse: GGAAGCTCCTCGATCTCTGA	Arginase-1	Forward: CCAGAAGAATGGAAGAGTCAGTGT Reverse: GCAGATATGCAGGGAGTCACC

Interleukin (IL)-10	Forward: AAGACCCAGACATCAAGGCG Reverse: AGGCATTCTTCACCTGCTCC	Fizz	Forward: TCCCAGTGAATACTGATGAGA Reverse: CCACTCTGGATCTCCCAAGA

IL-12 p35	Forward: GCCACAGGTCTGCATCCA Reverse: GACCTGGCGGGCTGAGTA	FcεRII	Forward: GGGACACAGCTCATGTTGGT Reverse: GCAGTGTCTCCCAGCTGTTT

IL-12 p40	Forward: AGCCTCCTCCTTGTGGCTA Reverse: TGGTTTTATCTTTTGTG TGTGC	IL-1β	Forward: GGTGTGTGACGTTCCCATTAG Reverse: TCGTTGCTTGGTTCTCCTTGT

iNOS	Forward: GTTCTCAAGGCACACCAGGTCTG Reverse: GCAGGTCACTTATGTCACTTATC	IL-12 p35	Forward: CTTAGCCAGTCCCGAAACCT Reverse: TTGGTCCCGTGTGATGTCT

MCP-1	Forward: GGAGCATCCACGTGTTGGC Reverse: ACAGCTTCTTTGGGACACC	IL-12 p40	Forward: GTTCAACATCAAGAGCAGTAGCA Reverse: CTGCAGACAGAGACGCCATT

MDC	Forward: GGTTGTCCTCGTCCTCCTTG Reverse: GAAGGTTAGCAACACCACGC	MCP-1	Forward: TCCCACTCACCTGCTGCTACTCA Reverse: GCTTCTTTGGGACACCTGCTG

MrC1	Forward: TCAATGGCATGAAGCGGAGA Reverse: TACTGTTCAGGGCGATCCAC	TNFα	Forward: ATGAGAAGTTCCCAAATGGC Reverse: CTCCACTTGGTGGTTTGCTA

18S	Forward: GGCCCTGTAATTGGAATGAGTC Reverse: CCAAGATCCAACTACGAGCTT	Yim-1	Forward: GGGCATACCTTTATCCTGAG Reverse: CCACTGAAGTCATCCATGTC

		Actin	Forward: GCCCTGAGGCTCTTTTCCAG Reverse: TGCCACAGGATTCCATACCC

### Immunoblot Analysis

Proteins were loaded into 10% polyacrylamide gels. Protein transferred to nitrocellulose membranes, which were immunoblotted with antibodies against AMPKα, p-AMPKα, ACC, p-ACC, NF-κB p65, p-NF-κB p65, ac-NF-κB p65, and SIRT1. Protein bands were visualized using the Supersignal West Dura Chemiluminescent Substrate (Thermo Fisher Scientific Inc., Rockford, IL, USA). Signals from bands were quantified using a UN-SCAN-IT gel™ software (Silk Scientific, Orem, UT, USA).

### RAN Interference Assay

For SIRT1 gene knockdown, cells were transfected with SIRT1 siRNA oligonucleotide (GenBank accession no. NM_019812.1: Daejeon, South Korea) using Lipofectamine 2000 (Invitrogen, Carlsbad, CA, USA) following the manufacturer’s protocol.

### Luciferase Assay

Cells were transiently transfected with NF-κB luciferase reporter vector using Lipofectamine 2000 (Invitrogen) according to the manufacturer’s instruction. The activity of firefly and luciferase was measured using the dual luciferase reporter assay system (Promega, Madison, WI, USA).

### Mice and Cell Culture

SIRT1-Tg mice (C57BL/6N) were a generous gift from Jong-Wan Park (Seoul National University, South Korea). C57BL/6N mice were from Japan SLC (Shizuoka, Japan). All experimental procedures were approved by the Animal Experimental Committee of the College of Medicine, Pusan National University (PNU-2016-1107) and done in accordance with guidelines for animal research. Murine bone marrow cells were isolated from mouse femoral and tibial bone marrow. Bone marrow-derived macrophages (BMDMs) were prepared from bone marrow cells and cultured in RPMI with 10% FBS and M-CSF (100 ng/ml).

### Induction and Monitoring of CIA

To trigger CIA, mice were sensitized by injecting 100 µg of chicken type II collagen (CII) supplemented with complete Freund’s adjuvant (Sigma) intradermally at the tail base, and received a booster injection of CII supplemented with incomplete Freund’s adjuvant in the same manner 14 days later by Inglis et al. (22). Mice were euthanized 38 days after initial injection, and knee joints were isolated. Arthritis severities in individual limbs were assessed by evaluating erythema, swelling, and other changes. Clinical arthritis severity and histological arthritis severity was scored using a scoring system, as previously described (23) (Table 2).

**Table 2:** Arthritis severity scoring system.

**Clinical arthritis severity score**	
0	Normal
1	Slight erythema or swelling
2	Distinct erythematous swelling
3	Joint distortion
4	Ankylosis of the joint
**Histological arthritis severity score**	
0	Normal
1	Synovial inflammation—mild Synovial lesion—mild alteration Cartilage destruction—mild Bone erosion—mild
2	Synovial inflammation—moderate Synovial lesion—moderate alteration Cartilage destruction—moderate Bone erosion—moderate
3	Synovial inflammation—moderate Synovial lesion—severe destruction of the synovia Cartilage destruction—severe destruction with loss of cartilage Bone erosion—severe destruction, with disrupted joint architecture

### Immunohistochemistry

Tissue sections were obtained from paraffin blocks and rehydrated, and then incubated with anti-TNFα, anti-IL-1β, and anti-TRANCE/TNFSF11 antibodies. Immunoreaction products were visualized using a broad-spectrum immunohistochemistry kit (Diaminobenzidine substrate kit, Vector Laboratories, Inc., Burlingame, CA, USA).

### Statistical Analysis

Statistical analyses were performed using GraphPad Software (San Diego, CA, USA). Means and SDs were calculated. The parametric Student’s *t*-test was used to assess the significances of differences between treated and untreated groups and considered to be significant when *P* < 0.05.

## Results

### Upregulation of M2 Markers by SIRT1 in RA Macrophages

Macrophages were generated from synovial monocytes cultured in the presence of M-CSF for 7 days (24). When cells were treated with RSV (a pharmacological activator of SIRT1, 50 µM) for 24 or 48 h, SIRT1 protein levels were significantly elevated (Figure S1 in Supplementary Material).

**Figure 1:**
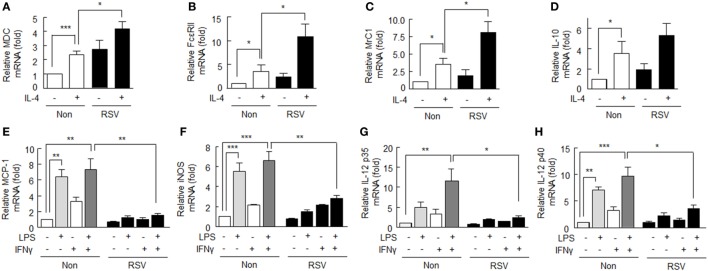
Effects of SIRT1 activation by resveratrol (RSV) on M1 and M2 markers in rheumatoid arthritis macrophages. **(A–D)** After pretreatment with RSV (50 µM) for 24 h, cells were incubated with interleukin (IL)-4 (20 ng/ml) for 12 h. The mRNA expression levels of MDC, FcεRII, MrC1, and IL-10 were quantified by qPCR and normalized versus 18S ribosomal RNA. **(E–H)** After pretreatment with RSV (50 µM) for 24 h, cells were incubated with LPS (1 µg/ml) and/or interferon (IFN)γ (20 ng/ml) for 12 h. The mRNA expression levels of MCP-1, iNOS, IL-12 p35, and IL-12 p40 were quantified by qPCR and normalized versus 18S ribosomal RNA. Values are means ± SEMs. Results are representative of four to six independent experiments. *P < 0.05, **P < 0.01, ***P < 0.001 as indicated.

To investigate the effect of SIRT1 on the M2 polarization of macrophages induced by IL-4 (20 ng/ml), we detected the mRNA levels of M2 macrophage markers, that is, MDC (macrophage-derived chemokine), FcεRII (low-affinity IgE receptors), MrC1 (C-type mannose receptor 1), and IL-10. As shown in Figures 1A–D, MDC, FcεRII, MrC1, and IL-10 mRNA expression levels following treatment with IL-4 (12 h) significantly increased to 2.37 ± 0.26-fold (*P* < 0.001), 3.41 ± 1.42-fold (*P* < 0.05), 3.53 ± 0.86-fold (*P* < 0.05), and 3.27 ± 1.17-fold (*P* < 0.05), respectively. When cells were pretreated with RSV (50 µM) for 24 h prior to IL-4, the mRNA expression levels of MDC, FcεRII, and MrC1 were significantly enhanced to 4.19 ± 0.52-, 10.73 ± 2.67-, and 8.10 ± 1.58-fold, respectively. IL-10 mRNA expression was only marginally increased.

### Suppres sion of Expression of M1 Markers by SIRT1 in RA Macrophages

M1 phenotype macrophages are promoted by macrophage-activating factors, such as LPS and IFNγ (25). Here, we examined whether SIRT1activation affects the LPS/IFNγ-induced M1 phenotype in RA. As shown in Figures 1E–H, M1 macrophage markers, including MCP-1, iNOS, and IL-12 p40 mRNA levels, were significantly elevated in response to LPS (1 µg/ml), but little affected by IFNγ (20 ng/ml) alone. However, the mRNA levels of MCP-1, iNOS, IL-12 p35, and IL-12 p40 were elevated further by LPS plus IFNγ. When cells were pretreated with RSV (50 µM) for 24 h prior to exposure to LPS plus IFNγ, the mRNA expression levels of M1 macrophage markers were significantly suppressed: MCP-1 (from 7.27 ± 1.43- to 1.56 ± 0.22-fold, *P* < 0.01), iNOS (from 6.63 ± 0.86- to 2.82 ± 0.33-fold, *P* < 0.01), IL-12 p35 (from 11.56 ± 2.98- to 2.43 ± 0.44-fold, *P* < 0.05), and IL-12 p40 (from 9.63 ± 1.70- to 3.56 ± 0.69-fold, *P* < 0.05), respectively.

These results show that SIRT1 activation by RSV augments the expression of anti-inflammatory M2 macrophages and attenuates the expression of pro-inflammatory M1 macrophages.

### Effects of SIRT1 Inhibition on M1/M2 Macrophages

**Figure 2:**
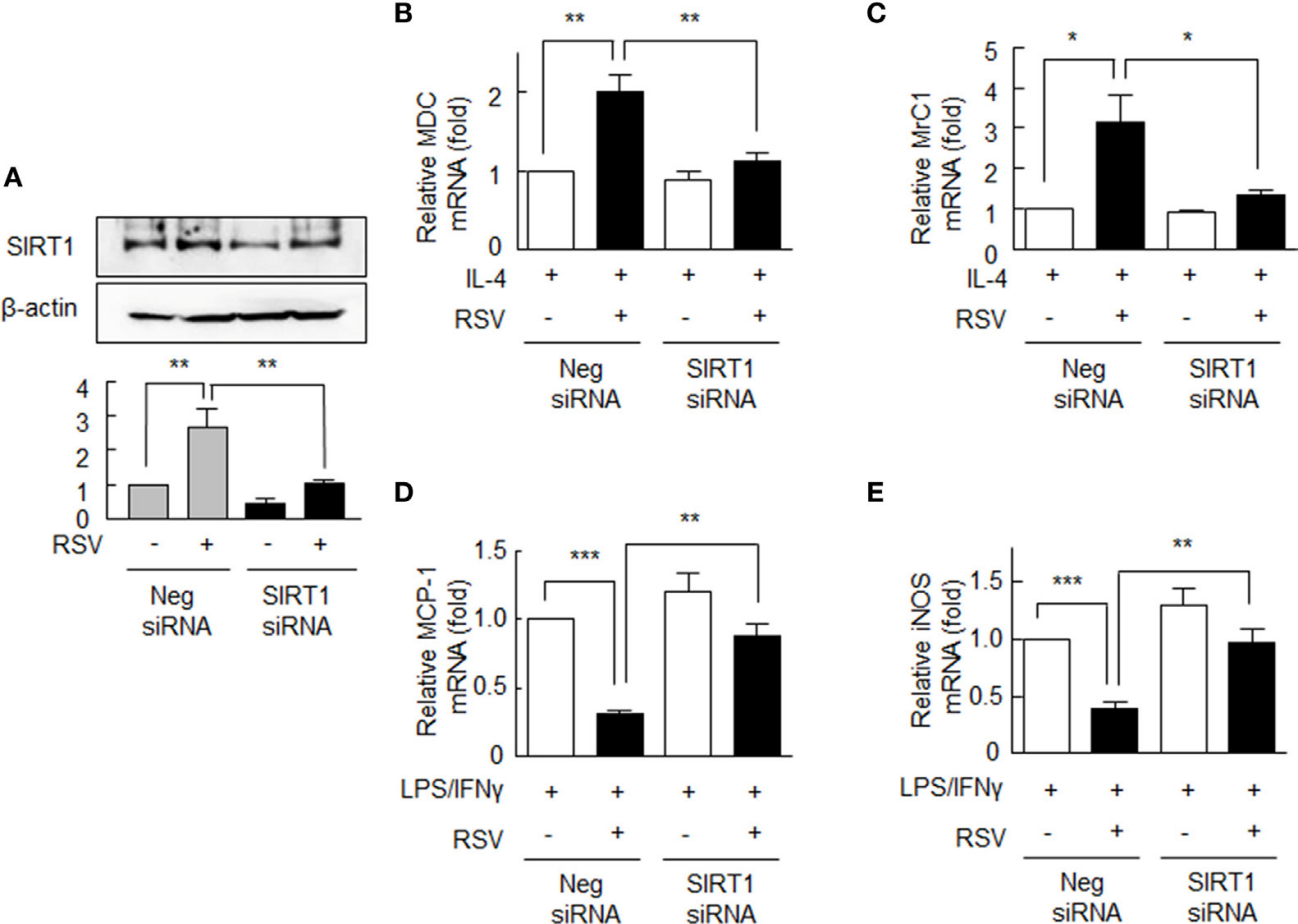
Expression of M1/M2 polarization markers in SIRT1 siRNA-transfected rheumatoid arthritis macrophages. **(A)** Cells were either transfected with the siRNA targeting SIRT1 (200 nM) or with scrambled siRNA duplex (200 nM) for 24 h and then were treated with or without resveratrol (RSV, 50 µM) for 24 h. **(B,C)** SIRT1 gene knockdown cells were pretreated with RSV (50 µM) for 24 h and were incubated with interleukin (IL)-4 (20 ng/ml) for 12 h, and the mRNA expression levels of MDC and MrC1 were quantified by qPCR. **(D,E)** These cells were incubated with LPS (1 µg/ml) and/or interferon (IFN)γ (20 ng/ml) for 12 h, and then the mRNA expression levels of MCP-1 and iNOS were quantified by qPCR. These levels were normalized versus 18S ribosomal RNA. Values are means ± SEMs. Results are representative of four independent experiments. *P < 0.05, **P < 0.01, ***P < 0.001 as indicated.

To confirm the role of SIRT1 in RA macrophage polarization into the M2 phenotype, SIRT1 expression in macrophages was inhibited by using of siRNA against SIRT1 gene. When cells were subjected to SIRT1 gene knockdown, they showed ~60% reduction in SIRT1 protein expression. These cells did not show increase in SIRT1 protein expression in response to RSV as contrasted to the cells transfected with negative siRNA (Figure 2A).

The mRNA levels of MDC and MrC1 were not elevated by RSV in cells transfected with SIRT1 siRNA, whereas in negative control cells MDC and MrC1 mRNA levels were significantly increased (Figures 2B,C). By contrast, in cells transfected with SIRT1 siRNA, RSV failed to suppress the mRNA levels of MCP-1 and iNOS in response to LPS plus IFN-γ, while both were significantly suppressed by RSV in negative control cells (Figures 2D,E). These results further support the notion that SIRT1 modulates the macrophage phenotype differentiation in RA.

### Induction of M2 Phenotype by Activation of SIRT1/AMPKα Phosphorylation

**Figure 3:**
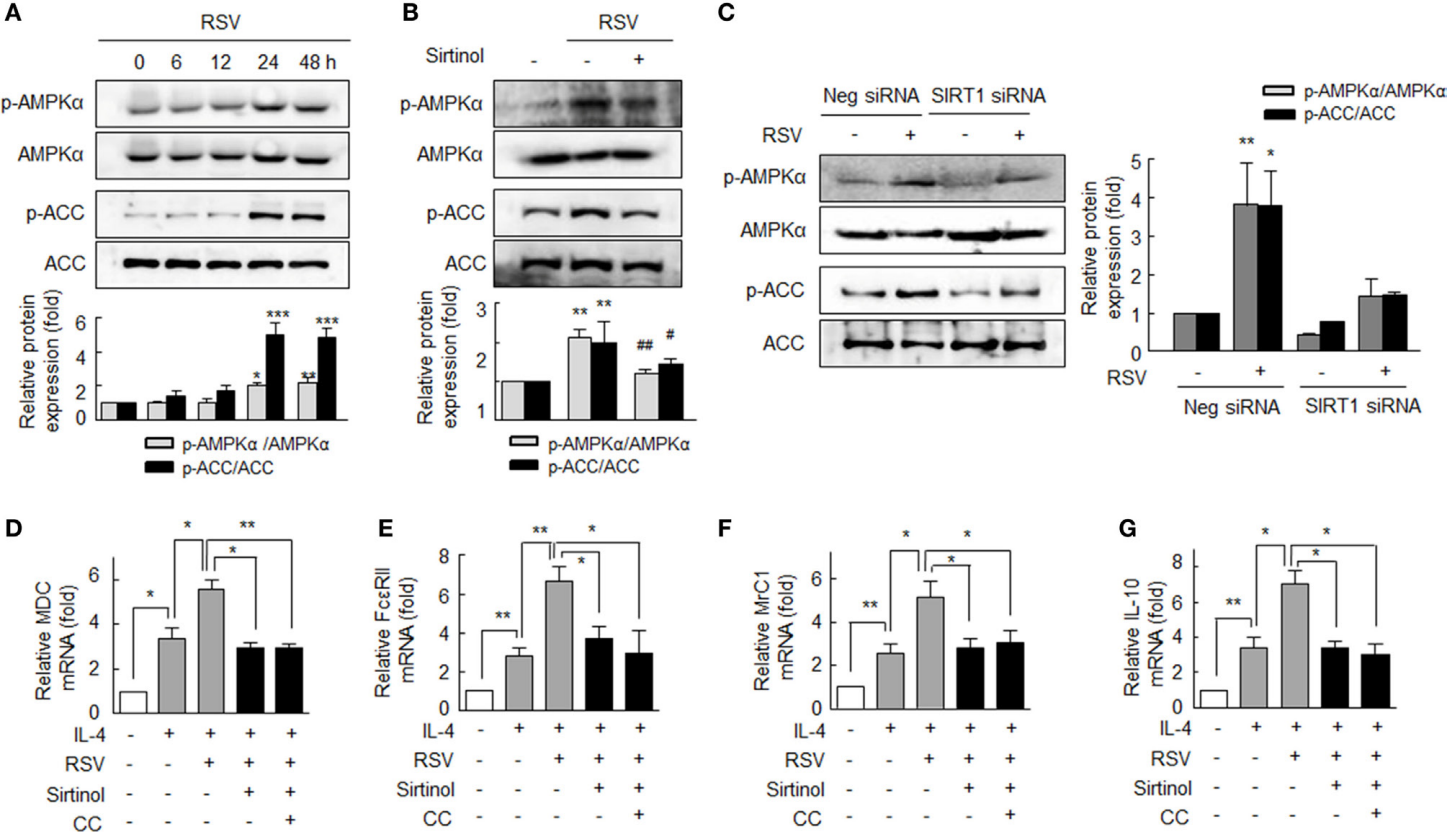
Enhanced macrophage M2 polarization by resveratrol (RSV) by SIRT1-mediated p-adenosine monophosphate-activated protein kinase α (AMPKα) upregulation. **(A)** Effects of SIRT1 on the phosphorylations of AMPKα and acetyl-CoA carboxylase (ACC). Cells were treated with RSV (50 µM) for 0–48 h. **(B)** The antagonizing effect of sirtinol (a SIRT1 inhibitor): cells were pretreated with sirtinol (20 µM) for 30 min and then with RSV (50 µM) for 24 h. **(C)** Effect of SIRT1 gene knockdown on the phosphorylations of AMPKα and ACC. SIRT1-gene knockdown cells were treated with RSV (50 µM) for 24 h. Values are means ± SEMs. Results are representative of four independent experiments. *P < 0.05, **P < 0.01, ***P < 0.001 versus zero time or none; ^#^*P* < 0.05, ^##^*P* < 0.01 versus RSV alone. **(D–G)** Inhibitions of resveratrol (RSV)-induced M2 macrophages markers by sirtinol and compound C (CC). After pretreatment with RSV (50 µM) for 24 h, cells were stimulated with interleukin (IL)-4 (20 ng/ml) for 12 h with or without sirtinol (20 µM) or CC (1 µM, an AMPK inhibitor) for 30 min. The mRNA expression levels of MDC, FcεRII, MrC1, and IL-10 were quantified by qPCR and normalized versus 18S ribosomal RNA. Values are means ± SEMs. Results are representative of four to five independent experiments. *P < 0.05, **P < 0.01 as indicated.

Based on a report that SIRT1 regulates AMPK signaling in attenuation of pro-inflammatory activity (10), we assessed whether the AMPK signaling activated by SIRT1 is linked to macrophage polarization to the M2 phenotype. To confirm that RSV activates AMPK in RA macrophages, we determined AMPKα phosphorylation at Thr^172^ (p-AMPKα) and ACC (a downstream target of AMPK) phosphorylation at Ser^79^ (p-ACC) (26). After treatment with RSV (50 µM for 24–48 h), p-AMPKα levels in cells were significantly increased, and similar results were obtained for p-ACC (Figure 3A). Furthermore, these increases were inhibited by pretreating RA macrophages with sirtinol (20 µM; a SIRT1 inhibitor) (Figure 3B). When cells were transfected with SIRT1 siRNA gene, RSV failed to increase the levels of p-AMPKα and p-ACC, whereas the expressions of both increased in negative control cells (Figure 3C).

We also investigated the involvement of the SIRT1/AMPKα signaling pathway in M2 macrophage polarization. As shown in Figures 3D–G IL-4 (20 ng/ml)-induced MDC, FcεRII, MrC1, and IL-10 mRNA expressions were significantly augmented by pretreating cells with RSV (50 µM). Interestingly, pretreatment with sirtinol (20 µM) or CC (1 µM, an inhibitor of AMPK) strongly prevented the RSV-induced augmentation of the expressions of MDC, FcεRII, MrC1, and IL-10 mRNAs, indicating that SIRT1/AMPKα signaling pathway leads to anti-inflammatory function of macrophages in RA.

### Suppression of M1 Macrophage Polarization by SIRT1 through Downregulating NF-κB

NF-κB activation has been reported to play a key role in LPS-induced M1 macrophage polarization, and M1 macrophages are known to have κB sites in their promoter regions, including those of CCL2, iNOS, and TNFα (27). Thus, we investigated the effect of RSV on the AMPKα activation under exposure to LPS plus IFNγ in RA macrophages. After pretreatment with RSV (50 µM) for 24 h, cells were incubated with LPS (1 µg/ml) plus IFNγ (20 ng/ml) for 0–48 h. We found intracellular levels of p-AMPKα and p-ACC had increased and that these increases were maintained after treatment for 48 h (Figure S2 in Supplementary Material).

**Figure 4:**
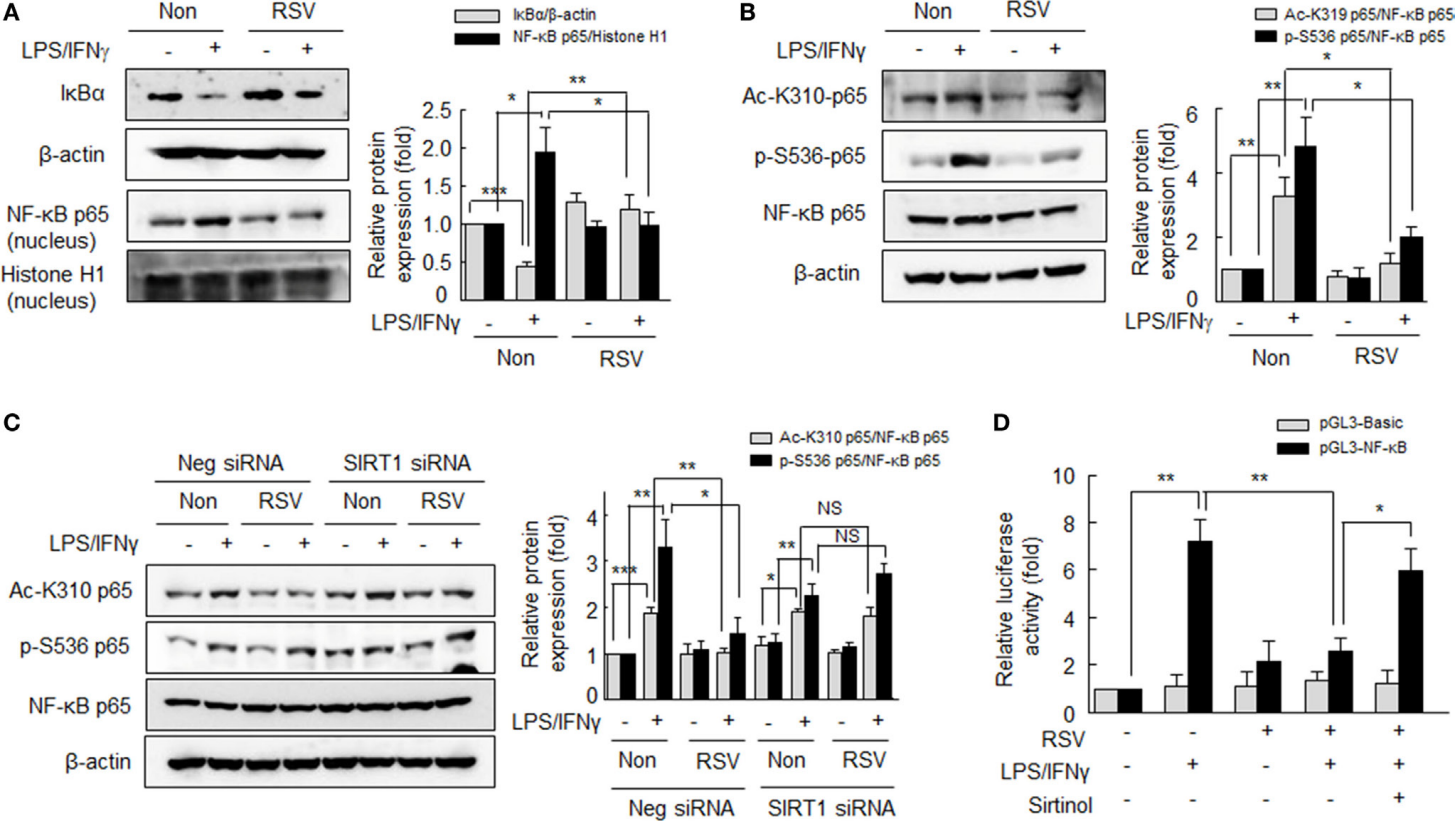
Suppression of NF-κB signaling by resveratrol (RSV) in the LPS/interferon (IFN)γ-induced M1 macrophage polarization. **(A)** Effects of RSV on IκBα degradation and nuclear NF-κB expression. After pretreatment with RSV (50 µM) for 24 h, cells were stimulated with LPS (1 µg/ml) plus IFNγ (20 ng/ml) for 1 h. **(B)** Effects of RSV on the acetylation of p65-K310 and the phosphorylation of p65-serine 536. Cells were pretreated with RSV (50 µM) for 24 h and then with LPS (1 µg/ml) plus IFNγ (20 ng/ml) for 1 h. **(C)** Effect of SIRT1 gene knockdown on NF-κB signaling. Cells were transfected with SIRT1 siRNA or negative control siRNA at a final concentration of 200 nM. Macrophages were incubated with RSV (50 µM) for 24 h, and then exposed to LPS (1 µg/ml) plus IFNγ (20 ng/ml) for 1 h. Values are means ± SEMs. Results are representative of four to five independent experiments.*P < 0.05, **P < 0.01, ***P < 0.001 as indicated. **(D)** Cells were transfected with basic-pGL3-luc or NF-κB-pGL3-luc using Lipofectamine 2000. After 24 h of transfection, cells were treated with or without sirtinol (20 µM) for 30 min, and then treated with RSV (50 µM) for 24 h followed by LPS (1 µg/ml) + IFNγ (20 ng/ml) for 3 h. The firefly luciferase enzyme activity were detected in cell extracts and normalized to the *Renilla* luciferase activity. Values are means ± SEMs. Results are representative of five independent experiments. *P < 0.05, **P < 0.01 as indicated.

To investigate the mechanism whereby SIRT inhibits polarization of macrophage to the M1 phenotype, we examined whether SIRT1 could inhibit NF-κB signaling. RA macrophages were pretreated with RSV (50 µM) for 24 h, and then further stimulated with LPS (1 µg/ml) plus IFNγ (20 ng/ml) for 1 h. As shown in Figure 4A LPS plus IFNγ significantly induced the degradation of IκBα in cytosol, whereas pretreatment of RSV resulted in prevention of the degradation of IκBα. In addition, RSV inhibited LPS plus IFNγ-induced nuclear translocation of NF-κB p65. Reportedly, NF-κB signaling is achieved through posttranslational modifications, such as phosphorylation and acetylation, which enhance the DNA-binding activity of p65 to the κB site (28–30). We then examined the effect of SIRT1 on NF-κB p65 acetylation on LPS plus IFNγ-induced M1 macrophage polarization. As shown in Figure 4B, increases in the levels of p65 acetylation at K310 and of p65 phosphorylation at Ser^536^ by LPS plus IFNγ were markedly inhibited by RSV pretreatment.

Furthermore, to define whether the prevention of NF-κB activity by RSV was mediated by SIRT1 activation, RA macrophages were subjected to SIRT1 knockdown by SIRT1 siRNA transfection. In the SIRT1 knockdown macrophages, the LPS plus IFNγ increased acetylation at K310 and phosphorylation of p65 at Ser^536^ were not suppressed by RSV, whereas they were significantly suppressed by RSV in negative control cells (Figure 4C). When cells were pretreated with RSV (50 µM) for 24 h prior to LPS plus IFN γ, increased NF-κB activity was attenuated and this reduction was prevented by sirtinol (20 µM) pretreatment (Figure 4D). These results suggest that SIRT1 prevents NF-κB p65 acetylation/phosphorylation, resulting in reduced M1 polarization.

### Reduced M1 Polarization in BMDMs from SIRT1 Tg-Mice

To determine the phenotypic characters of macrophages, we isolated BMDMs from SIRT1 Tg-mice and determined SIRT1 protein and AMPKα phosphorylation levels in these cells.

**Figure 5:**
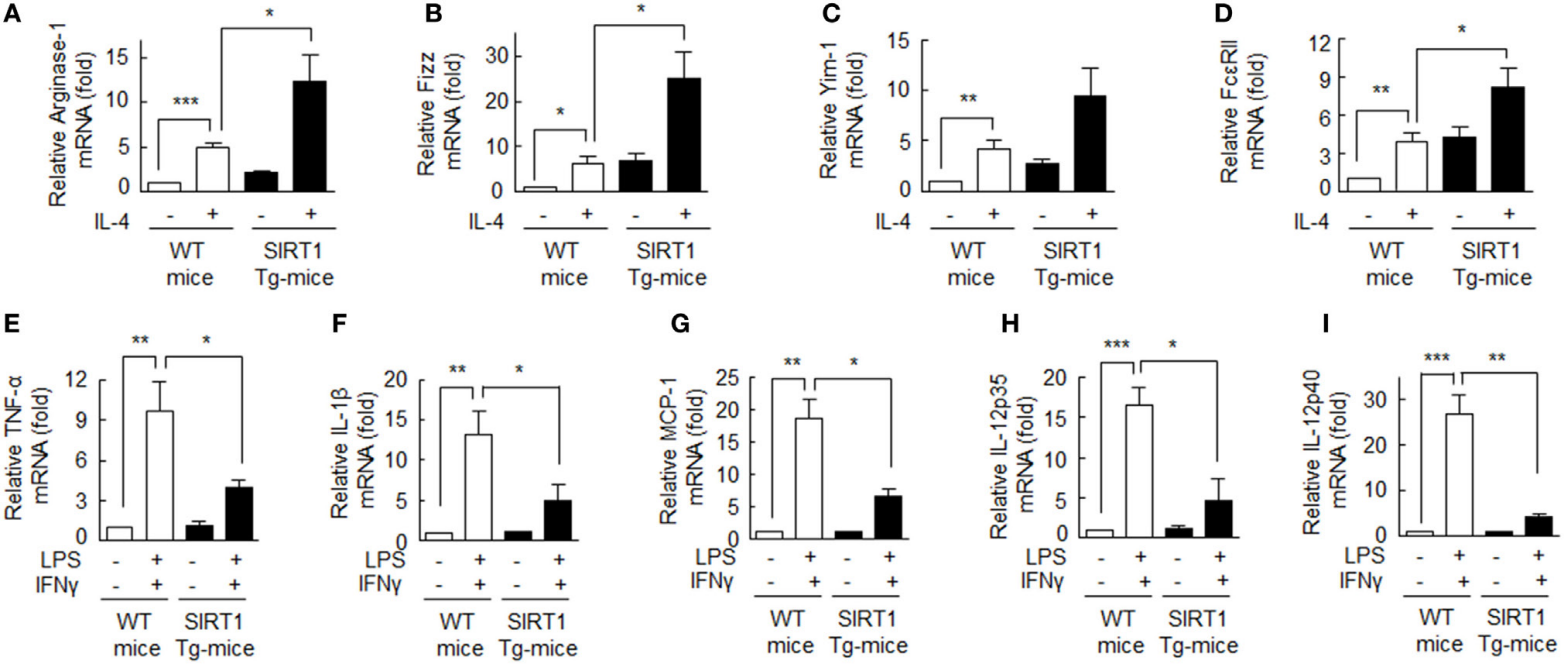
Upregulation of the M2 phenotype and downregulation of the M1 phenotype in bone marrow-derived macrophage (BMDMs) obtained from SIRT1 transgenic (Tg)-mice. **(A–D)** Cells were incubated with interleukin (IL)-4 (20 ng/ml) for 12 h and ariginase-1, Fizz, Yim-1, and FcεRII mRNA levels were then quantified with qPCR and normalization versus 18S ribosomal RNA. **(E–I)** Cells were incubated with LPS (1 µg/ml) or interferon (IFN)γ (20 ng/ml) for 12 h and TNFα, IL-1β, MCP-1, IL-12 p35, and IL-12 p40 mRNA levels were quantified by qPCR and normalization versus 18S ribosomal RNA. Values are means ± SEMs. Results are representative of five independent experiments. *P < 0.05, **P < 0.01, ***P < 0.001 as indicated.

Levels of SIRT1 protein and p-AMPKα were significantly higher in BMDMs from SIRT1 Tg-mice when compared with those from wild type (WT) mice (Figure S3 in Supplementary Material). BMDMs from SIRT1 Tg-mice were treated with IL-4 (20 ng/ml), and the mRNA levels of M2 macrophage markers, including ariginase-1, Fizz, Yim-1, and FcεRII were assessed. As shown in Figures 5A–D, the degree of expressions of ariginase-1, Fizz, Yim-1, and FcεRII mRNAs were significantly higher in response to IL-4 in macrophages obtained from SIRT1 Tg-mice than those from WT controls, suggesting increased SIRT1/AMPK levels prominently promote M2 macrophage polarization.

By contrast, when BMDMs from SIRT1 Tg-mice were incubated with LPS (1 µg/ml) plus IFNγ (20 ng/ml), mRNA levels of the M1 phenotype markers: TNF-α, IL-1β, MCP-1, IL-12 p35, and IL-12 p40 were significantly suppressed (Figures 5E–I), indicating that SIRT1 strongly suppressed M1 macrophage activation. These results confirm SIRT1 promotes macrophage polarization toward the M2 phenotype.

### Attenuation of Inflammatory Joint Disease Induction by SIRT1 in CIA Mice

**Figure 6:**
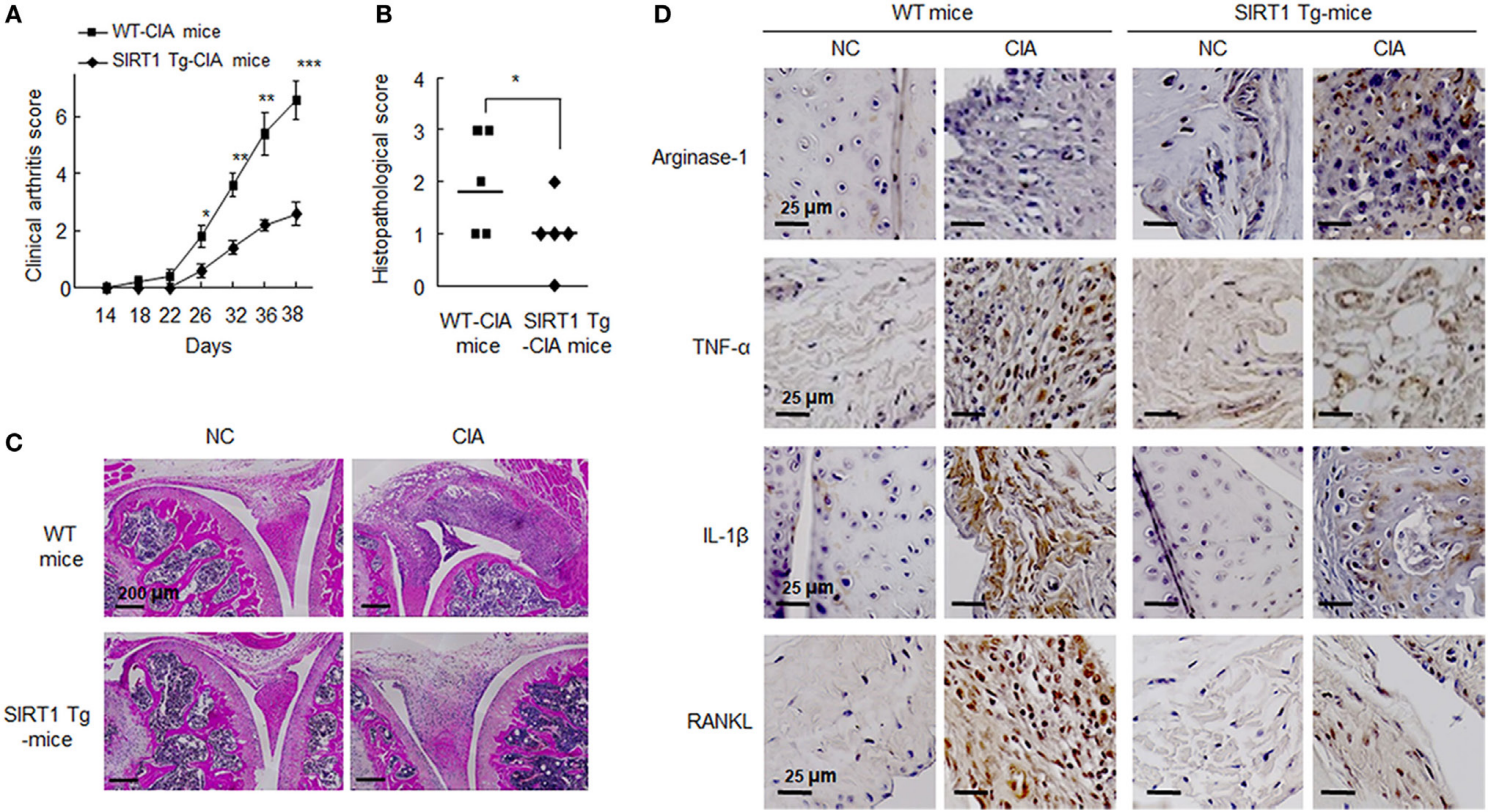
Analysis of the pathological severity of the joints of C57BL/6N and SIRT1 transgenic (Tg) mice (*n* = 5) subjected to collagen-induced arthritis (CIA) (refer to Section “Materials and Methods” for details). **(A)** Arthritis severity scores of CIA mice were recorded after second immunization with type II collagen. **(B)** Histological scores of synovitis, pannus formation, and erosion in knee joints. Values are means ± SEMs. Results are representative of five independent experiments. *P < 0.05, **P < 0.01, ***P < 0.001 versus SIRT1 Tg-CIA mice. **(C)** Representative H&E staining of joints from CIA mice. Scale bar = 200 µm. **(D)** Representative sections showing staining for arginase-1, TNF-α, interleukin (IL)-1β, and RANKL, which were markedly reduced in the knee joint tissues of SIRT1 Tg-CIA mice. Tissue sections from knee joints were stained with the indicated antibodies. Staining for each antibody on each cell are shown in dark brown. Scale bars; 25 µm.

The mouse CIA model was employed because it is similar immunologically and pathologically to RA patients. SIRT1-Tg CIA mice developed less severe arthritis than WT CIA mice, as evidenced by lower scores of disease activity from days 26 to 38 (Figure 6A). On day 38, the mean clinical arthritis score of SIRT1 Tg-CIA mice (2.6 ± 0.4) was significantly lower than that of WT CIA mice (6.6 ± 0.68) (Figure 6B).

On day 38 after first injection, knee joints from SIRT1-Tg CIA and WT CIA mice were obtained for microscopic analysis. They were blindly scored for histological signs of arthritis, that is, cartilage destruction, bone erosion, and cell infiltration. Histological articular damage was significantly less severe in the knee joints of in SIRT1 Tg-CIA mice than in those of WT CIA mice (Figure 6C). Histological images indicated that synovial hyperplasia, bone and cartilage destruction, vascular proliferation, and inflammatory cells infiltration were markedly diminished in SIRT1-Tg CIA mice.

In addition, we assessed the expression of M2 marker arginase-1, M1 marker TNFα, and IL-1β in the knee joints by immunohistochemistry. Ariginase-1 marker in SIRT1-Tg mice was detected relatively higher in SIRT1-Tg mice than WT mice, and SIRT1 Tg-CIA mice showed less decrease in ariginase-1 expression. In contrast to ariginase-1, TNFα and IL-1β were rarely detected in SIRT1 Tg-CIA mice, compared to WT CIA mice. A similar result was obtained for RANKL expression (Figure 6D). These results indicate the SIRT1 overexpression leads to anti-inflammatory responses in association with suppression of synovial inflammation, joint destruction in the murine CIA model.

## Discussion

The current study shows when RA macrophages were treated with IL-4, SIRT1 enhances macrophage polarization into the M2 phenotype by upregulating the phosphorylations of AMPKα and ACC. In line with these findings, SIRT1 significantly suppressed the M1 phenotype polarization of macrophages by inhibiting NF-κB activation. In addition, we observed that expressions of TNFα and IL-1β, the pro-inflammatory cytokines, were significantly lower in SIRT1 Tg-CIA mice than in WT CIA mice, and these reductions were associated with the suppression of synovial inflammation and bone destruction. These findings support the notion that the anti-inflammatory responses in RA are induced by activation of SIRT1/AMPKα signaling pathways.

Kennedy et al. (31) have emphasized that synovial macrophages exert a crucial role in the pathogenesis of inflammation in RA, by initiating and resolving inflammation by generating various cytokines. Macrophages populations are heterogeneous and the extents of these heterogeneities are dependent on microenvironmental signals. Dysregulation of M1/M2 polarization in macrophages was known to reflect the pathogenesis of inflammation (32). Moreover, Hah et al. (33) have advised SIRT1 as a potential therapeutic target to treat inflammatory arthritis, based on the facts that SIRT1 lack myeloid cells exacerbated inflammation due to the NF-κB hyperactivation and increased productions of cytokines associated with M1 polarization.

SIRT1 regulates the functions of several important transcription factors with anti-inflammatory effects (21, 33). However, no direct evidence supported the role of SIRT1 in macrophage polarization. In view of these situations, we focused on the negative regulatory role of SIRT1 in RA inflammation (21). M2-polarized macrophages are characterized by the upregulations of mannose receptor, CD23, CCL22, and scavenger receptor, which all have anti-inflammatory effects (34). In this study, SIRT1 activation by RSV in RA macrophages enhanced the IL-4-induced M2 phenotype, whereas treatment with SIRT1 inhibitor suppressed anti-inflammatory response. Intriguingly, the present results showed that overexpression of SIRT1 in macrophages induced an anti-inflammatory state by inducing genes encoding M2 markers, such as arginase-1, Fizz, Yim, and FcεRII. In addition, SIRT1 also inhibited M1 macrophage polarization. The expressions of M1 marker genes induced by LPS/IFNγ stimulation (a well-known means of inducing M1 polarization) were reduced in SIRT1-activated macrophages, and the high expressions of M1 markers in macrophages from WT mice treated with LPS/IFNγ were markedly lower in macrophages from SIRT1 Tg-mice. These findings suggest that SIRT1 might be useful for ameliorating RA-associated inflammation.

It has been argued SIRT1 aggravates inflammation by upregulating the expressions of pro-inflammatory cytokines in synovial fibroblasts (19) and in the synovial tissues of smokers with RA (35). Nevertheless, Wendling et al. (17) have revealed that SIRT1 expression and its activity in the cytoplasm of PBMCs from RA patients are lower than in healthy controls. Recently, we reported PMA-induced NF-κB transcriptional activation and secretions of pro-inflammatory cytokines (TNFα, IL-1β, and IL-6) were suppressed by RSV treatment, and NF-κB transcriptional activity was more largely suppressed in association with reduction in TNFα, IL-1β, and IL-6 mRNA and protein levels in the SIRT1 transgenic mice compared to the control C57BL/6 mice (21). Sag et al. (36) further emphasized that AMPK counteracts inflammatory signalings in macrophages by inhibiting LPS-induced IκBα degradation and by upregulating Akt/CREB activation. They suggested that AMPK directs signaling pathways in macrophages to suppress pro-inflammatory responses and promote macrophage polarization toward an anti-inflammatory phenotype. Similarly, in chronic kidney disease patients, the upregulation of AMPK activation leads to polarization toward the M2 phenotype in accompanying with the restoration of mitochondrial biogenesis in macrophages (37). Our present results showed SIRT1 activation by RSV increased AMPKα phosphorylation and ACC phosphorylation in RA macrophages, but not in SIRT1 gene knockdown RA macrophages. In line with these findings, the pharmacological inhibitors sirtinol (a SIRT1 inhibitor) and CC (a chemical inhibitor of AMPK) both significantly blocked SIRT1-stimulated increases in M2 macrophage polarization.

We found that SIRT1 strongly suppressed p65 acetylation at K310 and phosphorylation at Ser^536^, and that SIRT1 increased IκBα expression in cytosol, and decreased the nuclear translocation NF-κB p65, and consequently decreased NF-κB to κB binding, which agrees with previous reports (12, 13). This result was further supported by the experiment with SIRT1 gene knockdown macrophages, in which LPS plus IFNγ increased NF-κB p65 acetylation at K310 and p65 phosphorylation at Ser^536^ were not suppressed by RSV, whereas both were significantly suppressed by RSV in negative control cells. These results are also supported by Yang et al. (15), in that AMPKα negatively regulates pro-inflammatory genes due to the deacetylation of NF-κB by SIRT1 in macrophages. These results indicate that SIRT1/AMPKα signaling enhanced M2 and suppressed M1 macrophage polarization by inhibiting NF-κB in RA macrophages.

In this study, the findings obtained from SIRT1-Tg CIA mice model supported the implication of SIRT1 in RA inflammation *in vivo*. In particular, histological articular damage was more attenuated in the knee joints of SIRT1-Tg CIA mice compared to WT CIA mice, and this reduction in articular damage was found to be associated with reduced synovial hyperplasia, bone and cartilage destruction, synovial angiogenesis, and inflammatory cell infiltration. In summary, our results show that the SIRT1/AMPKα signaling pathway is involved in the control of macrophage polarization and that SIRT1 probably acts as a negative regulator of the inflammatory processes associated with RA. We conclude that SIRT1 modulation offers a promising strategy for treating RA inflammation.

## Ethics Statement

All experimental procedures were approved by the Animal Experimental Committee of the College of Medicine, Pusan National University (PNU-2016-1107) and done in accordance with guidelines for animal research.

## Author Contributions

SYP designed and did the major experiments, analyzed the experimental data, and contributed to the writing. KWH and CDK designed and contributed to the writing. SWL and SYL designed the experiments and analyzed the experimental data. SSB and KK did the critical revision of the manuscript.

## Conflict of Interest Statement

The authors declare no potential conflicts of interest.
