# Supplementary material for: SIRT1/Adenosine Monophosphate-Activated Protein Kinase α Signaling Enhances Macrophage Polarization to an Anti-inflammatory Phenotype in Rheumatoid Arthritis
Source: Front Immunol. 2017 Sep 15;8:1135. doi: 10.3389/fimmu.2017.01135 (PMC5605563; doi:10.3389/fimmu.2017.01135)
Supplement: Supplementary file 1 [file Data_Sheet_1.DOCX]

**Supplemental Data**

**MATERIALS AND METHODS**

**Antibodies**

Anti-AMPKα, anti-phosphorylated-AMPKα (Thr 172) (p-AMPKα), anti-acetyl-CoA carboxylase (ACC), and anti-p-ACC (Ser 79) antibodies were purchased from Cell Signaling (Danvers, MA), and anti-SIRT1 was from Santa Cruz Biotechnology Inc. (Santa Cruz, CA).

**Western Blot Analysis**

Proteins (30 μg) were loaded onto 10% SDS-polyacrylamide electrophoresis gels, electrophoresed, and transferred to nitrocellulose membranes (Amersham Biosciences, Inc., Piscataway, NJ), which were then immunoblotted using antibodies against AMPKα, p-AMPKα, ACC, p-ACC, and SIRT1. Immunoblots were visualized by chemiluminescence using the Supersignal West Dura Extended Duration Substrate Kit (Pierce Chemical, Rockford, IL). Signals from bands were quantified using a calibrated imaging densitometer (GS-710; Bio-Rad, Hercules, CA).

**Statistical Analysis**

The analysis were performed using GraphPad Software (San Diego, CA). Means and standard deviations were calculated. The Student’s t-test was used to determine the significances of differences between groups. P values less than 0.05 were considered significant.

**Supplemental Figure 1**

Effect of resveratrol on SIRT1 expression in RA macrophages.

Cells were treated with resveratrol (50 μM) for 0-48 h, and cell lysates were immunoblotted with anti-SIRT1 antibody. Results are represented as means ± SEMs of four independent experiments. ****P* < 0.001 vs. none; ^#^*P* < 0.05, ^##^*P* < 0.01 vs. control.

**Supplemental Figure 2**

Effect of LPS/IFN-γ on the resveratrol-induced phosphorylations of AMPKα and ACC.

After pretreatment with resveratrol (50 μM) for 24 h, cells were incubated with LPS (1 μg/ml) + IFN-γ (20 ng/ml) for 0-48 h. Results are represented as means ± SEMs of duplicates each pooled four independent experiments.

**Supplemental Figure 3**

Expression of SIRT1 and phosphorylation of AMPKα in BMDM of SIRT1 Tg-mice.

Cells were lysed, and the immunoblotted using the indicated antibodies. ****P* < 0.001 vs. none; ^#^*P* < 0.05, ^##^*P* < 0.01 vs. BMDMs from WT-mice.
