# Supplementary figures and images for: SIRT1/Adenosine Monophosphate-Activated Protein Kinase α Signaling Enhances Macrophage Polarization to an Anti-inflammatory Phenotype in Rheumatoid Arthritis
Source: Front Immunol. 2017 Sep 15;8:1135. doi: 10.3389/fimmu.2017.01135 (PMC5605563; doi:10.3389/fimmu.2017.01135)

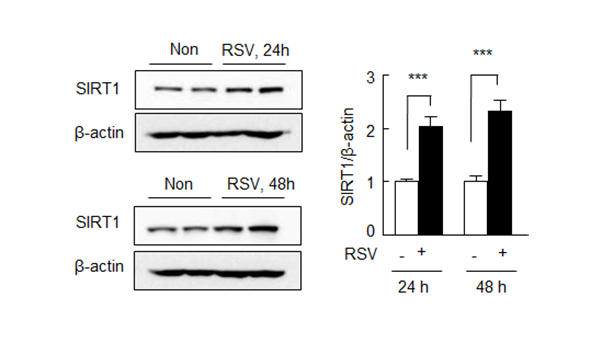

Supplement: Supplementary file 2 [file Image_1.TIF]

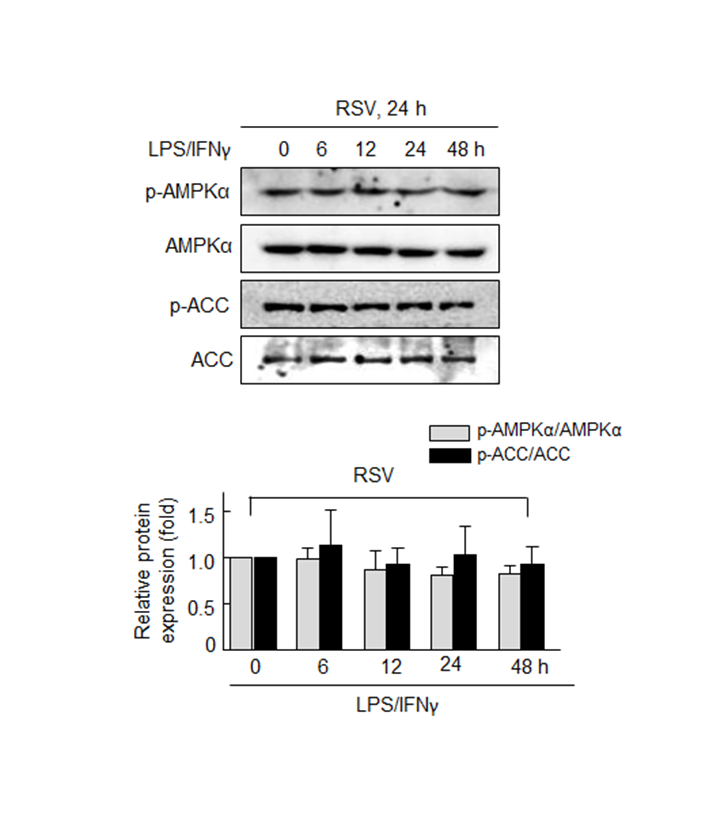

Supplement: Supplementary file 3 [file Image_2.TIF]

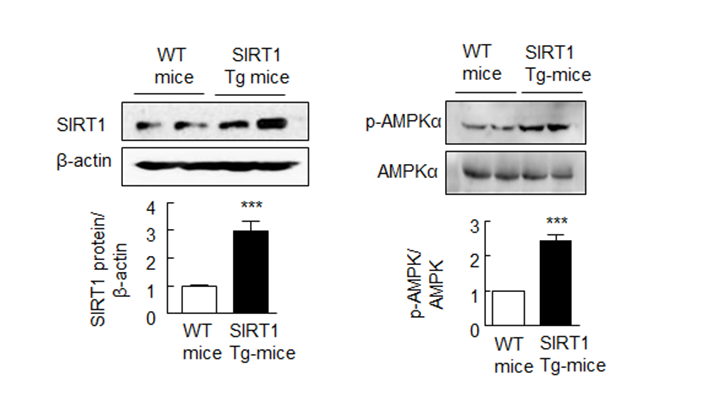

Supplement: Supplementary file 4 [file Image_3.TIF]
